# Atypical connectivity in the cortico-striatal network in NF1 children and its relationship with procedural perceptual-motor learning and motor skills

**DOI:** 10.1186/s11689-022-09428-y

**Published:** 2022-03-01

**Authors:** Eloïse Baudou, Federico Nemmi, Patrice Peran, Fabien Cignetti, Melody Blais, Stéphanie Maziero, Jessica Tallet, Yves Chaix

**Affiliations:** 1 ToNIC, Toulouse NeuroImaging Center, University of Toulouse, Inserm, UPS, Toulouse, France; 2 Children’s Hospital, Toulouse-Purpan University Hospital, Toulouse, France; 3 Pediatric Neurology Unit, Hôpital des Enfants, CHU Toulouse, 330 av de Grande Bretagne-TSA, 31059 Toulouse, France; 4 CNRS, TIMC-IMAG, Université Grenoble Alpes, Grenoble, France; 5 EuroMov Digital Health in Motion, Université Montpellier, IMT Mines Ales, Montpellier, France

**Keywords:** Procedural memory, Serial reaction time task, Neurofibromatosis type 1, Resting-state MRI, Cortico-striatal connectivity, Neurodevelopmental disorder

## Abstract

**Introduction:**

Neurofibromatosis type 1 (NF1) is considered a model of neurodevelopmental disorder because of the high frequency of learning deficits, especially developmental coordination disorder. In neurodevelopmental disorder, Nicolson and Fawcett formulated the hypothesis of an impaired procedural learning system that has its origins in cortico-subcortical circuits. Our aim was to investigate the relationship between cortico-striatal connectivity and procedural perceptual-motor learning performance and motor skills in NF1 children.

**Methods:**

Seventeen NF1 and 18 typically developing children aged between 8 and 12 years old participated in the study. All were right-handed and did not present intellectual or attention deficits. In all children, procedural perceptual-motor learning was assessed using a bimanual visuo-spatial serial reaction time task (SRTT) and motor skills using the Movement Assessment Battery for Children (M-ABC). All participants underwent a resting-state functional MRI session. We used a seed-based approach to explore cortico-striatal connectivity in somatomotor and frontoparietal networks. A comparison between the groups’ striato-cortical connectivity and correlations between connectivity and learning (SRTT) and motor skills (M-ABC) were performed.

**Results:**

At the behavioral level, SRTT scores are not significantly different in NF1 children compared to controls. However, M-ABC scores are significantly impaired within 9 patients (scores below the 15th percentile). At the cerebral level, NF1 children present a higher connectivity in the cortico-striatal regions mapping onto the right angular gyrus compared to controls. We found that the higher the connectivity values between these regions, differentiating NF1 and controls, the lower the M-ABC scores in the whole sample. No correlation was found for the SRTT scores.

**Conclusion:**

NF1 children present atypical hyperconnectivity in cortico-striatal connections. The relationship with motor skills could suggest a sensorimotor dysfunction already found in children with developmental coordination disorder. These abnormalities are not linked to procedural perceptual-motor learning assessed by SRTT.

**Supplementary Information:**

The online version contains supplementary material available at 10.1186/s11689-022-09428-y.

## Background

Neurofibromatosis type 1 (NF1) is one of the most common childhood autosomal dominant neurogenetic disorders, affecting 1 in 2500 to 3000 individuals [1]. Learning deficits are frequent and represent the main neurological complication of the disease [2]. Several neurodevelopmental disorders may be encountered in NF1, such as developmental coordination disorder (DCD), dyslexia, specific language impairment, or attention deficit/hyperactivity disorder [3].

Nicolson and Fawcett proposed the hypothesis that children with learning deficits have impairments of the procedural learning system [4]. This system subserves the learning of sensorimotor and cognitive skills, rules, and habits [5]. In their model, the procedural learning system (PLS) is divided into a cortico-striatal PLS and a cortico-cerebellar PLS, which are dissociated in the language and motor systems. The motor cortico-striatal PLS is said to be impaired in DCD, and the language cortico-cerebellar PLS in dyslexia. In this study, we will be particularly interested in the motor cortico-striatal PLS, given that about 50% of NF1 children meet the criteria for DCD [6].

The serial reaction time task (SRTT) is the most common task for assessing procedural perceptual-motor learning [7]. In adults with NF1 without cognitive or neurological impairments, using an established form of non-invasive, double-pulse transcranial magnetic stimulation (dp-TMS) during a finger-tapping task with the non-dominant hand, Zimerman et al. found a substantial decline in motor skill learning linked to an intracortical inhibition, especially after the early acquisition phase [8].

Studies of procedural learning in children are sparse. Studies using SRTT to assess procedural learning in neurodevelopmental disorders such as DCD found either preserved learning [9, 10] or impaired learning in a bimanual condition [11]. Both adults and children with alterations in the basal ganglia present deficits in procedural perceptual-motor learning. This is the case in Parkinson’s or Huntington’s diseases [12, 13] and in children with acquired alteration in the striatum, regardless of the etiology of the lesion and the age of occurrence [14].

Human brain imaging studies have demonstrated that the cortico-striatal networks are involved in perceptual-motor sequence learning, while the cerebello-cortical networks are involved in motor adaptation [15–17]. Motor procedural learning is a process requiring repetition and time to automatize movement. It can be broken down into three steps: fast learning, slow learning allowing consolidation and automatization, and retention. The striatum is the main brain area involved in motor sequence learning [16]. Functional brain imaging studies have shown that activations during a motor sequence learning task are focused at first on the associative striatum but shift to the sensorimotor striatum with practice. The cortical areas involved in the cortico-striatal circuit are the motor cortical regions, the parietal cortices, the frontal associative region, and the medial temporal lobe (hippocampus). Recent results suggest that children with developmental coordination disorders present a dysfunction in the cortico-striatal network with overconnectivity in the frontoparietal cortico-striatal networks linked to the score on the Movement Assessment Battery for Children (M-ABC) [18]. Such a dysfunction may be linked to procedural perceptual-motor deficit. In NF1 children, significant differences have been reported with higher volumes, lower fractional anisotropy, and higher mean diffusion in the striatum, which includes the putamen and the caudate nucleus [19]. These brain areas are also prone to present unidentified bright objects (UBOs), which are frequently reported (in 2/3 of NF1 children), as well as metabolic abnormalities in spectroscopic MRI [20]. The presence of these abnormalities is an indication of axonal damages associated with an increase in myelin turnover in areas of intramyelinic edema [21, 22]. Given that NF1 children have both striatal abnormalities and frequent associated bimanual coordination disorders [23], one could suggest a possible impairment of procedural perceptual-motor learning using a bimanual SRTT.

Resting-state functional MRI has been used to better understand the connectivity in patients with basal ganglia diseases (Parkinson’s disease [24], Huntington’s disease [25]) using a seed-to-voxel analysis based on the functional atlas of the striatum. This imaging analysis has also been used in typically developing children [26] and in neurodevelopmental disorders [18]. In NF1 patients, neuroimaging studies have reported abnormal connectivity. Tomson reported reduced anterior-posterior connectivity and altered modularity clustering in NF1 adults relative to healthy controls [27]. Using a voxel-to-voxel analysis, Nemmi et al. found a higher local correlation in NF1 children compared to TD, specifically in the right superior temporal gyrus, the right middle frontal gyrus, and the right cuneus [28]. On the other hand, clusters in the left and right frontal poles, the left inferior frontal gyrus, the left insula, the left parahippocampal cortex, and the bilateral precuneus and cingulate cortex showed higher local correlation for typically developing children (TD) relative to NF1. Until now, an investigation into the connectivity of striatal subregions in NF1 patients has not been reported.

The aim of this study is to explore the functional connectivity in the cortico-striatal pathways and its relationship with motor abilities and procedural perceptual-motor learning in NF1 children.

## Methods

### Participants

This study is part of the DYSTAC-MAP protocol. Seventeen children with NF1 and eighteen TD participated in the study. All were right-handed and aged from 8 to 12 years old. The two groups did not differ in age or sex.

The children did not present a known neurological or psychiatric disorder, uncorrectable hearing or visual impairment, intellectual disability (WISC total IQ superior to 70 and/or subtest similarities and picture concept scores greater than 7), or ADHD criteria (DSM-5 diagnostic criteria). All children underwent motor skills assessment using the Movement Assessment Battery for Children (M-ABC) in its French translation [29]; a score below the 15th percentile was an exclusion criterion for the control group. Children with NF1 had been diagnosed with NF1 by a pediatric neurologist, in accordance with the Neurofibromatosis Conference statement (1988) [30]. Demographic, psychomotor, and neuropsychological characteristics are presented in Table 1. The study was approved by the local ethics committees and was conducted in accordance with the Declaration of Helsinki. We obtained written informed consent from the children and their parents.

**Table 1:** Demographic and psychomotor characteristics of NF1 and typically developing children (TD)

	NF1 (***n*** = 17)	TD (***n*** = 18)	Statistic	***p***-value
**Demographic characteristics**				
Mean age in months (SD; range)	116 (20.23; 96–171)	121 (14.65; 96–150)	117.0	0.241
Sex ratio (male/female)	0.41	0.55	131.0	0.413
**Psychomotor characteristics**				
Mean M-ABC score (SD)	16.07 (19.56)	50.51 (26.35)	40.0	< 0.001

Note that NF1 children had significantly lower M-ABC scores, and nine of them had scores below the 15th percentile. The Mann-Whitney *U* test was used for the M-ABC score, as it was not normally distributed. The results were presented as means (standard deviation)

### Behavioral tasks

We used a classical visuospatial SRTT to assess procedural perceptual-motor memory and implicit learning [7]. A computer with a 24-in. screen was placed 80 cm in front of the participant. A yellow square successively appeared at one of four positions arranged horizontally 3 cm apart from each other on a black computer screen. Each screen position corresponded to a button on a response pad: D, F, G, or H. The participant was asked to press the corresponding button as quickly as possible with the index or middle finger of each hand (bimanual responses). As soon as a response was given or after a time of 3000 ms without a response, the next stimulus appeared after a time interval of 250 ms. The task consisted of a sequence of ten visual stimuli that were repeated ten times to form a block (1 block = 100 stimuli). The task was composed of 6 blocks. The first four blocks (B1–B4) and the last one (B6) included the repeated sequence. The fifth block (B5) was a pseudorandomized block in which the visual cue no longer played out a repeating pattern of positions. The participant was not advised that the visual cues followed a repeated sequence of positions, which made learning implicit.

Motors skills were assessed using the total M-ABC score. The M-ABC is a test of motor impairment developed and validated for use with children ages 4–12 years old divided into four age categories (4–6 years old, 7–8 years old, 9–10 years old, 11–12 years old). It includes eight items that assess both fine and gross motor skills divided into three categories: manual dexterity, balance, and ball handling. The sum of the score items gives a deterioration score that is transformed and reported here, as age-related percentiles.

### MRI acquisition

MRI images were acquired using a Philips Achieva dStream 3.0-T MRI scanner equipped with a 32-channel head coil. Rs-fMRI: echo-planar imaging (EPI) sequence. Time repetition (TR)/time echo (TE) = 3000/40 ms, flip angle (FA) = 90°, field of view (FOV) = 240 mm, matrix = 80 × 80, voxel size = 3.0 × 3.0 × 3.0 mm, 46 axial slices. Each scan session was 600 s long and included 200 volumes. Resting-state data were acquired while each subject was asked to stay awake, eyes opened, and not thinking about anything in particular.

### MRI processing

Rs-fMRI images were preprocessed using the Conn software including the following steps: realignment and unwarp, slice timing correction, and outlier detection using strict settings: 97th percentiles in the normative sample, global signal *z*-value threshold 5, subject motion mm threshold 0.9, direct segmentation and normalization (also applied to T1 images for each subject), smoothing with an 8-mm kernel. BOLD time series were denoised using the aCompCor method [31–33] of the CONN toolbox (www.nitrc.org/projects/conn; [34]), which consisted in regressing out from the functional time-series the first two principal components of the time-series extracted from white matter and CSF. The BOLD time series were finally band-pass filtered (0.008–0.09 Hz).

### Functional connectivity

A seed-to-voxel analysis was performed using the seven-network striatal atlas [35]. This atlas consists of the parcellation of the human striatum into 1000 subjects, based on functional connectivity to seven major networks: sensorimotor, ventral attention, dorsal attention, frontoparietal, default, visual, and limbic networks. We restricted our analysis to the sensorimotor (“striatal seed 2” of Choi’s atlas) and frontoparietal (“striatal seed 6” of Choi’s atlas) networks based on previous results [18]. Using GLM analysis, we first analyzed the maps of connectivity in all participants to compare the profiles of connectivity obtained in our sample with those from the literature [18, 35].

### Statistical analysis

Data were analyzed using the JAMOVI (1.6.15.0) software [36] or the GLM analysis option of the Conn toolbox.

#### Comparison of the behavioral task between groups

For the procedural perceptual-motor memory assessment, we performed a repeated measures ANOVA on reaction times between B1 and B4 (general learning), B4 and B5 (specific learning), and B5 and B6 (retention), with the group as the intersubject factor and the blocks as intrasubject factors. The normality of data was assessed by the Shapiro-Wilk test, the homogeneity of variance with Levene’s test, and the sphericity with Mauchly’s test. As the variable “number of errors” for each block was not normally distributed, we used a non-parametric Friedman test on each group with the blocks as intrasubject factors for general learning and a paired sample *t* test for specific learning and retention.

To compare the performance on motor skills in the two groups, we used the Mann-Whitney *U* test, because the distribution of variables assessed by the Shapiro-Wilk test was not normal. For each analysis, the *p*-value was set at *p* < 0.05.

#### Comparison of functional connectivity between the groups

We explored the group differences between the NF1 and TD groups, including the following covariables: age, sex, and GCOR (average global correlation). All analyses were thresholded by applying the cluster-forming threshold *p* < 0.001 and the cluster-extent threshold P-FDR < 0.05.

#### Correlation between functional connectivity values and behavioral scores

Correlations were performed between the connectivity values, expressing a significant difference between NF1 and TD children and [1] learning SRTT scores, [2] the M-ABC score, and then [3] between SRTT scores and M-ABC score. Correlations were performed using Pearson or Spearman correlations, depending on the normal distribution of variables assessed by the Shapiro-Wilk test. Bonferroni corrections for multiple comparison corrections had been done. Learning scores were calculated for reaction times, i.e., the difference in the results between B1 and B4 for general learning, between B4 and B5 for specific learning, and between B5 and B6 for retention.

## Results

### SRTT

**Fig. 1:**
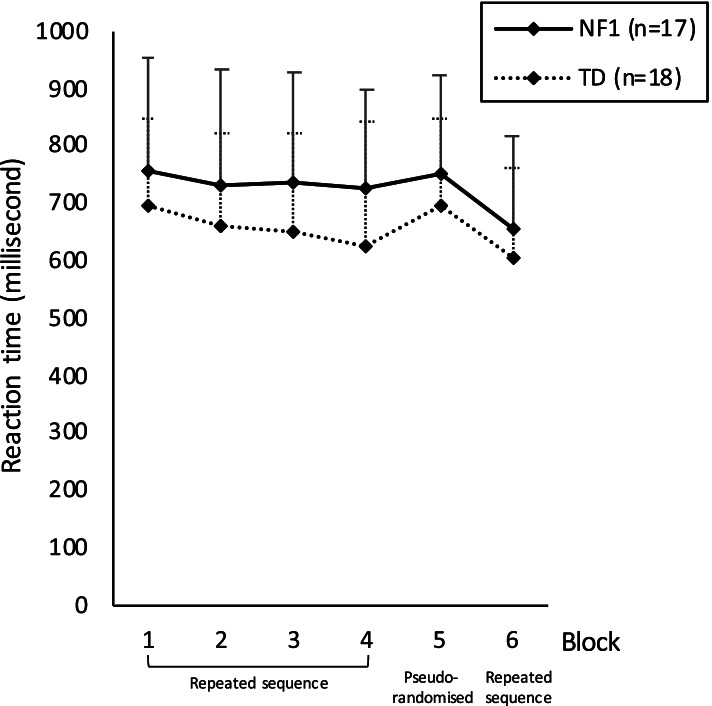
Serial reaction time performances in neurofibromatosis type 1 (NF1) and typically developing children (TD). Vertical bars represent interindividual variability (standard errors)

The results of the SRTT are presented in Fig. 1.

The repeated ANOVA measures showed a block effect, but no group effect or interaction. There was a significant difference between B1 and B4 (*F* (3.08) = 52.01; *p* = 0.015) with better performance with practice, as well as between B5 and B4 (*F*(− 2.85) = − 48.05; *p* = 0.019) with lower performance in the random block representing specific learning and between B5 and B6 (*F* (5.45) = 92.128; *p* < 0.001) with better performance on repeated sequences than on the pseudorandomized sequence, expressing retention of the repeated sequence. There were no significant results concerning the number of errors in each group.

### Cortico-striatal estimated functional network

**Fig. 2:**
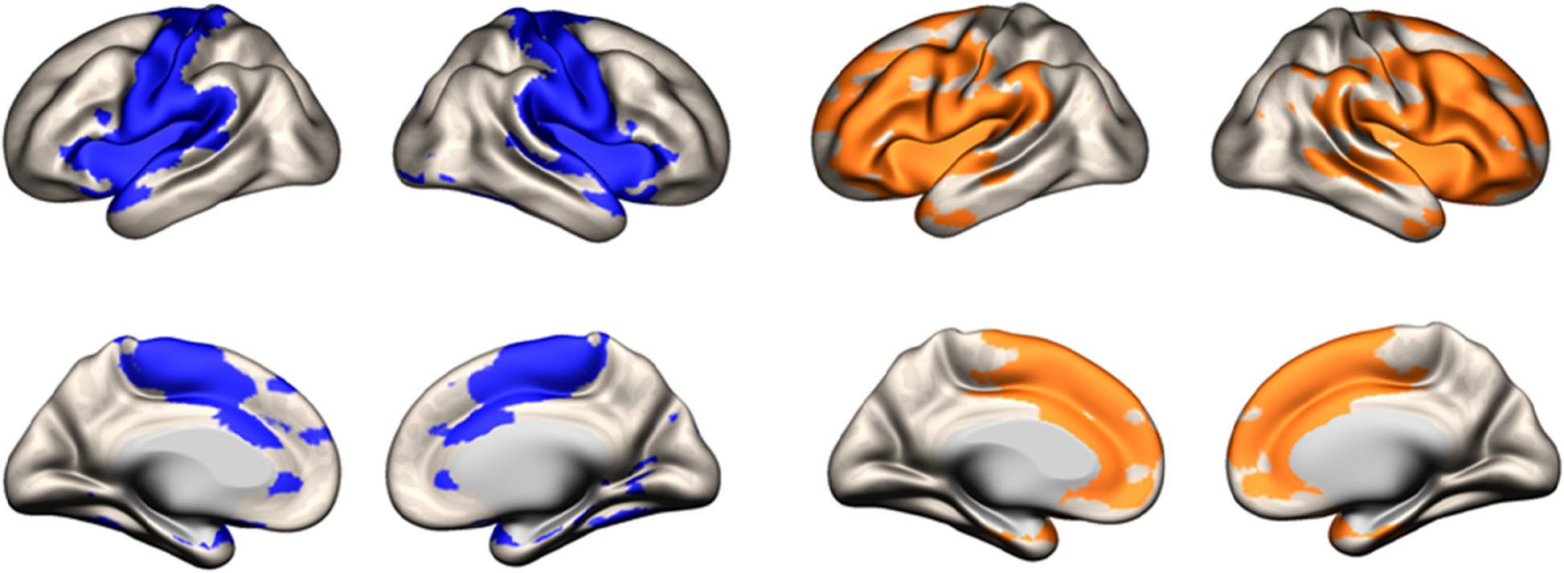
Areas showing significant positive connectivity with the striatal seeds in the somatomotor network (in blue) and in the parietofrontal network (in orange) (*p* < 0.001, family-wise error-corrected, cluster size > 100)

The estimated topography of somatomotor and parieto-frontal networks in our child groups (Fig. 2) was qualitatively closed to topography previously found in adults [35] and children [19] with connection respectively with primary motor, somatosensory cortices, and prefrontal and posterior parietal cortices.

### Impact of NF1 on the cortico-striatal functional circuit

**Fig. 3:**
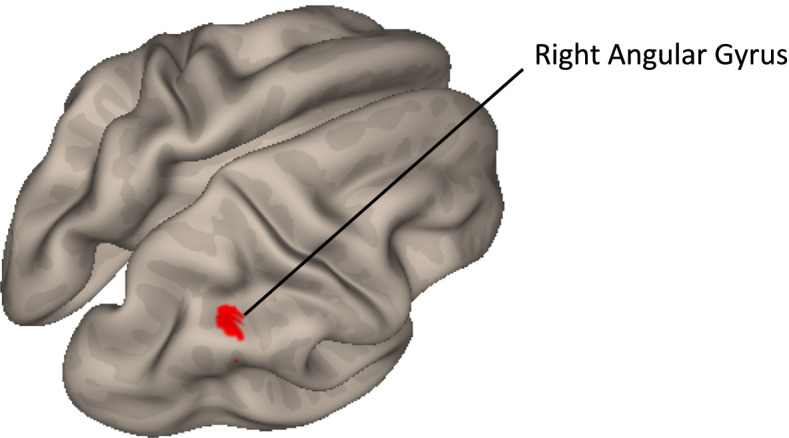
Cerebral regions of the cortico-striatal functional circuits that expressed any difference between NF1 and typically developing children. The right angular gyrus (AG) is significantly more connected with the somatomotor striatal seed “striatum 2” of Choi’s functional atlas in NF1 children than in typically developing children. Age, sex, and GCOR are used as covariables; voxel threshold *p* < 0.001 *p*-uncorrected and cluster threshold *p* < 0.05

Cortico-striatal connectivity significantly differed between NF1 and controls in a 148-voxel region (MNI coordinates, + 36 − 50 + 30) in the right angular gyrus connected with the somatomotor striatal seed (Fig. 3).

### Relationship between connectivity in the cerebral regions of the cortico-striatal functional circuits that expressed a significant difference between NF1 and TD children and behavioral scores

**Fig. 4:**
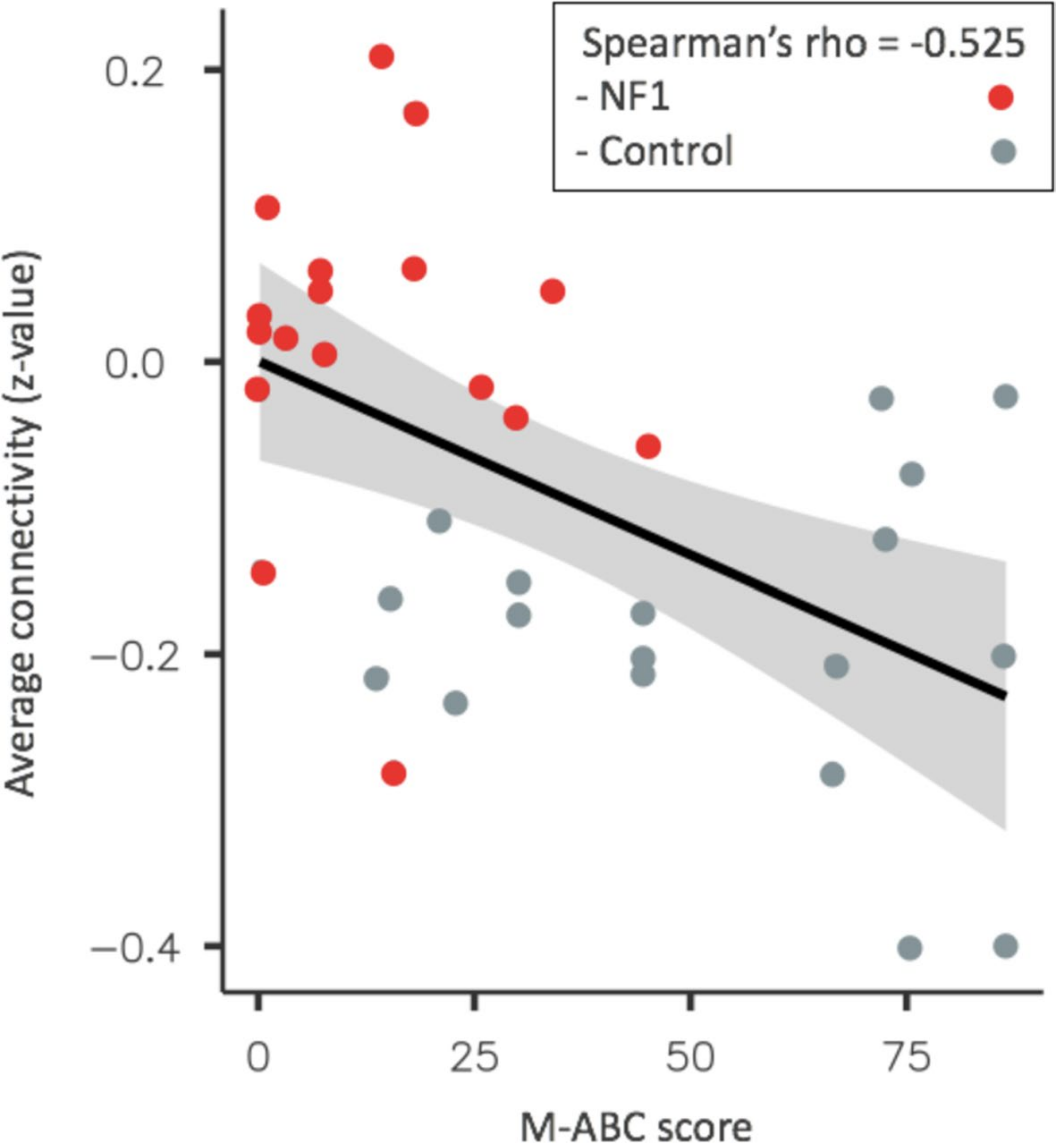
Correlation between the M-ABC score and the average connectivity values within a significant cluster between the NF1 and TD groups

No correlation was found between SRTT scores and the average connectivity values between the second striatal seed and the right angular gyrus cluster. Moreover, there was no correlation between learning scores in SRTT and M-ABC scores. However, as shown in Fig. 4, the average connectivity significantly decreased as the M-ABC score increased (Spearman’s rho = − 0.525; *p*-value < 0.001; Bonferroni correction applied). That is to say that children with lower motor skills had a higher connectivity score between the somatomotor striatal seed and the right angular gyrus cluster differentiating the NF1 and TD groups. In the NF1 group, 11 children have positive connectivity values while in the TD group, all participants have negative connectivity values.

## Discussion

The aim of this study was to explore the functional connectivity in the cortico-striatal pathways and its relationship with motor abilities and procedural perceptual-motor learning in NF1 children. We found that NF1 children present higher connectivity in the cortico-striatal regions mapping onto the right angular gyrus compared to controls and a relationship between this hyperconnectivity and motor skills but not with procedural learning. The results are discussed in terms of abnormal connectivity of the angular gyrus in NF1 children, the absence of correlation between learning scores and this abnormal connectivity, and the heterogeneous performance of NF1 children at the end of learning (specific learning and retention).

The angular gyrus is located in the posterior part of the inferior parietal lobule. This area is involved in numerous functions, and it is considered a cross-modal hub where converging multisensory information is combined and integrated to comprehend and give sense to events, manipulate mental representations, solve familiar problems, and reorient attention to relevant information [37]. Numerous studies have evaluated the link between the angular gyrus and perceptual-motor sequential learning. Using gray matter density, volumes, or cortical thickness analyses, studies have shown structural plasticity in the angular gyrus when healthy adults learn new skills relative to spatial coordination, verbal storage, and creativity [38–40]. Moreover, functional MRI studies during SRTT in healthy adults have reported activations in the motor and premotor cortices [41–44], the parietal cortex [41–45], and the basal ganglia [41, 42, 44–46]. However, a recent neuroanatomical metanalysis of neural correlates of learning with SRTT showed that if visual and motor parameters were correctly controlled, specific sequence learning yielded consistent activation only in the basal ganglia, across the striatum (anterior/mid-caudate nucleus and putamen), and in the globus pallidus [16]. Cerebellar and premotor regions appear to contribute to the aspects of the task not related to learning of the sequence itself: executive, working memory, or other attention-related functions for cerebellar regions and sensorimotor integration for premotor regions.

Based on the hypothesis of an alteration in procedural learning in neurodevelopmental disorders, we explored whether the difference in cortico-striatal connectivity could be linked with scores in a visuo-spatial sequence learning task. This relationship was not found in our study. Firstly, at a behavioral level, NF1 children did not have significantly lower results in the different SRTT scores, suggesting no deficit in procedural learning of perceptual-motor sequences. Thus, the hyper-connectivity in the cortico-subcortical circuit in NF1 could be viewed as a compensatory mechanism allowing them to learn as well as controls but at the cost of increased connectivity. However, the absence of a relationship between the learning score and hyper-connectivity does not allow this hypothesis to be validated. In fact, hyperconnectivity in the cortico-subcortical circuit in NF1 seems to be a characteristic of patients with neurodevelopmental disorders presenting sensorimotor dysfunction. Cignetti et al. showed that intrinsic cortico-subcortical functional connectivity is affected in children with developmental coordination disorder (DCD), including abnormalities in the cortico-striatal connections mapping onto the posterior parietal cortex (angular gyrus and supramarginal gyrus), not present in developmental dyslexia [18]. Cerliani et al. also reported resting-state hyper-connectivity in the cortico-subcortical pathways targeting sensorimotor regions (both the cerebellum and the basal ganglia) in male subjects from 7 to 50 years with autism spectrum disorder (ASD) [47]. The strength of the interaction between networks was associated with age and autistic traits indexed by the Social Responsiveness Scale. The authors suggested that this association is compatible with the idea of a relationship between sensory abnormalities and repetitive behaviors in ASD patients. Motor skills were not reported in this study, but the results suggest that hyper-connectivity in the cortico-subcortical pathways is particularly associated with atypical sensorimotor behaviors, which are found in some NF1 children.

Several hypotheses can be made to explain the absence of a correlation between behavior and brain connectivity. Firstly, SRTT is a task exploring only a part of procedural memory involving visuo-spatial motor sequence learning in an early stage. It would be interesting to explore the long-term retention, consolidation, and automatization of SRTT and their relations with the hyperconnectivity in the cortico-subcortical pathways. Secondly, we selected NF1 patients without intellectual disability and ADHD in this study, which represents about 5 and 30% of NF1 children, respectively. This strict exclusion criterion allows motion-induced artifacts to be prevented in our analyses and focalization on the motor sequence learning process, excluding conditions which themselves contribute to the difficulties in learning but limit the generalization of our results to the whole NF1 population. However, the NF1 phenotype is quite heterogeneous, and great variability had been shown both in cognitive results and in brain characteristics [19]. In line with these results, it is interesting to note that NF1 children presented a heterogeneous performance at the end of learning (see errors during blocks 4, 5, and 6 that correspond to specific learning and retention in Supplementary data), which could suggest that a subgroup of NF1 children present a learning deficit. Further studies with larger samples are required to explore this heterogeneity. Lastly, functional connectivity differences linked with procedural learning between NF1 children and typically developing children may be a dynamic process that could not be highlighted at rest but only when children were scanned while performing the task.

The level of motor skills does not seem to be the best candidate to predict the level of procedural learning. Indeed, our results failed to find a relationship between the learning scores and the level of motor skills using the total M-ABC score. The SRTT is a short learning task that assesses the first step of learning (fast learning) and not consolidation, long-term retention, and other cognitive and motor processes such as motor adaptation, visuo-spatial abilities [48], global coordination of the whole body, or manual dexterity that is needed in perceptual-motor skills already consolidated, assessed in the M-ABC. It is possible that the absence of correlation between these two scores comes from a discrepancy between the ability to improve motor skills (SRTT) and the ability to perform them (M-ABC). A source of heterogeneity, in our NF1 population, is that nine children met the criteria for DCD (particularly, M-ABC score < 5th percentile). Another source of heterogeneity is the possible presence of UBOs (abnormalities in myelination) appearing in 75% of children in the first two years of life and disappearing at the end of adolescence [49]. UBOs are considered a radiologic marker of demyelination. While some studies have found a correlation between thalamo-striatal UBOs and cognitive deficit (lower IQ and visuospatial performances) [50], most of them have failed or results have not been reproduced, making the real impact of UBOs on cognitive functions unclear. However, the impact of UBOs specifically on procedural learning had not been studied. As children with acquired brain lesions in the basal ganglia had altered sequence learning scores [14], we can question the impact of UBOs, which can be considered a marker of dynamic alteration of the brain microstructure in NF1 children. Unfortunately, in our functional MRI study, the impact of UBOs on cortico-striatal connectivity is not evaluable because of the small sample size (only six NF1 patients presented UBOs in the basal ganglia in our study).

## Conclusion

NF1 children present overconnectivity in the cortico-striatal connections mapping onto the right angular gyrus, linked to M-ABC scores but not with SRTT scores. These results reinforce the importance of considering NF1 a neurodevelopmental disorder, given that the same pattern of results has been found in children with developmental coordination disorder. Such a modification of the cortico-striatal connectivity has been found in other neurodevelopmental disorders with atypical sensorimotor behaviors (e.g., ASD), with the hypothesis of a loss of GABAergic projection neurons within the striatum. Future studies using magnetic resonance spectroscopic and functional MRI examination during SRTT could improve our understanding of the involvement of the cortico-striatal circuit in procedural learning in neurodevelopmental disorders.

## Supplementary Information

**Additional file 1: Supplementary data**.

## Data Availability

The datasets analyzed during the current study are available from the corresponding author on reasonable request.
