# Supplementary material for: Atypical connectivity in the cortico-striatal network in NF1 children and its relationship with procedural perceptual-motor learning and motor skills
Source: J Neurodev Disord. 2022 Mar 1;14:15. doi: 10.1186/s11689-022-09428-y (PMC8903485; doi:10.1186/s11689-022-09428-y)
Supplement: Supplementary file 1 — Additional file 1: Supplementary data. [file 11689_2022_9428_MOESM1_ESM.docx]

Supplementary data

|  | ***NF1 (n=17)*** | ***TD (n=18)*** | ***Statistics*** |  |
| --- | --- | --- | --- | --- |
| **Mean** **Reaction Times (ms) (SD)** | | | | |
| Block 1 | 757.00 (SD:151.97) | 695.41 (SD:196.02) | Repeated ANOVA measures  Main block effect  B1-B4 p=0.015  B4-B5 p=0.019  B5-B6 p<0.001  No group effect. No interaction | |
| Block 2 | 729.46 (SD:160.70) | 658.19 (SD:203.50) |  |  |
| Block 3 | 737.18 (SD:174.05) | 648.91 (SD:189.78) |  |  |
| Block 4 | 723.67 (SD:216.28) | 624.72 (SD:173.77) |  |  |
| Block 5 | 749.92 (SD:152.68) | 694.63 (SD:174.09) |  |  |
| Block 6 | 656.13 (SD:155.39) | 604.17 (SD:161.04) |  |  |
| **Mean Number of Errors (SD)** | | | | |
| Block 1 | 4.18 (SD:2.811) | 5.28 (SD:3.86) | In each group:  Non-parametric Friedman test for B1-B4 Paired sample t-test for B4-B5 and B5-B6  No significant results | |
| Block 2 | 5.41 (SD:4.26) | 4.94 (SD:4.30) |  |  |
| Block 3 | 5.41 (SD:3.76) | 6.40 (SD:5.18) |  |  |
| Block 4 | 7.47 (SD:9.27) | 6.00 (SD:4.97) |  |  |
| Block 5 | 9.59 (SD:8.70) | 8.00 (SD:4.92) |  |  |
| Block 6 | 7.59 (SD:8.70) | - 1. SD:6.24) |  |  |
